# Clinical and Economic Burden of Community-Acquired Pneumonia among Adults in the Czech Republic, Hungary, Poland and Slovakia

**DOI:** 10.1371/journal.pone.0071375

**Published:** 2013-08-06

**Authors:** Ales Tichopad, Craig Roberts, Igor Gembula, Petr Hajek, Anna Skoczynska, Waleria Hryniewicz, Karina Jahnz-Rozyk, Roman Prymula, Ivan Solovič, Vitězslav Kolek

**Affiliations:** 1 CEEOR spol. s r.o., Prague, Czech Republic; 2 Emerging Markets Business Unit, Pfizer Inc., Collegeville, Pennsylvania, United States of America; 3 Department of Epidemiology and Clinical Microbiology, National Medicines Institute, Warsaw, Poland; 4 Department of Immunology & Allergology, Military Institute of Health Service, Warsaw, Poland; 5 University Hospital Hradec Kralove, Hradec Kralove, Czech Republic; 6 I. Department of Pneumology and Ftizeology, National Institute for Tuberculosis, Lung Diseases and Thoracic Surgery, Vysne Hagy, Slovakia; 7 Department of Pulmonary Diseases and Tuberculosis, University Hospital Olomouc, Olomouc, Czech Republic; Health Protection Agency, United Kingdom

## Abstract

We estimate and describe the incidence rates, mortality, and cost of CAP (community-acquired pneumonia), in both inpatient and outpatient settings, in the Czech Republic (CZ), Slovakia (SK), Poland (PL), and Hungary (HU). A retrospective analysis was conducted on administrative data from the health ministry and insurance reimbursement claims with a primary diagnosis of pneumonia in 2009 to determine hospitalization rates, costs, and mortality in adults ≥50 years of age. Patient chart reviews were conducted to estimate the number of outpatient cases. Among all adults ≥50 years, the incidence of hospitalized CAP per 100,000 person years was: 456.6 (CZ), 504.6 (SK), 363.9 (PL), and 845.3 (HU). The average fatality rate for all adults ≥50 is 19.1%, and for each country; 21.7% (CZ), 20.9% (SK), 18.6% (PL), 17.8% (HU). Incidence, fatality, and likelihood of hospitalization increased with advancing age. Total healthcare costs of CAP in EUR was 12,579,543 (CZ); 9,160,774 (SK); 22,409,085 (PL); and 18,298,449 (HU); with hospitalization representing over 90% of the direct costs of treatment. The burden of CAP increases with advancing age in four CEE countries, with hospitalizations driving the costs of CAP upwards in the elderly population. Mortality rates are generally higher than reported in Western EU countries.

## Background

Lower respiratory tract infections, including pneumonia, are the third leading cause of death worldwide, responsible for an estimated 3.8 million deaths in 2008.[1] Community-acquired pneumonia (CAP) is an acute disease which represents a common cause of hospital admission and mortality in developed countries and hence consumes a great proportion of health care budgets. The most recent guidelines of The European Society of Clinical Microbiology and Infectious Diseases define suspected community-acquired pneumonia (CAP) as: “An acute illness with cough and at least one of new focal chest signs, fever >4 days or dyspnoea/tachypnea, and without other obvious cause”. Definite confirmation of CAP is then described as: “Above but supported by chest radiograph findings of lung shadowing that is likely to be new. In the elderly, the presence of chest radiograph shadowing accompanied by acute clinical illness (unspecified) without other obvious cause.[2]” Empirical guidelines for treatment of pneumonia have been proposed in Europe and in the USA.[3]–[6]

Hospitalized CAP is associated with a high mortality in the elderly, and the incidence rate and mortality of CAP increases with increasing age and the presence of comorbidity.[7]–[13] CAP presents a considerable risk of early mortality, even in low-risk patients [11], and is associated with excess mortality beyond the period of the initial episode [14].

In Europe, pneumonia costs have been estimated at €10.1 billion annually, with inpatient care accounting for €5.7 billion, outpatient care €0.5 billion, and drugs €0.2 billion. The indirect costs of lost work days are estimated to be €3.6 billion in 2003 [15]. Ongoing cost-containment efforts have shifted the provision of care to the outpatient settings, and only those with most severe disease and multiple comorbid illnesses are admitted to hospitals. A substantial portion of all CAP cases are hence treated as outpatients and frequently not reported to national databases. This causes a systematic underestimation of the complete burden of the disease. In the USA, the percentage of elderly adults with CAP treated as outpatients was reported as 59.3% in 2004 [12]. In parallel, stratification of patients by severity of disease and treatment to established guidelines has enabled reductions in hospital length of stay (LOS) and better identification of patients who can be treated in an ambulatory setting [13].

It is known that CAP incidence, mortality, and cost vary with age, gender, and comorbidity and that the mortality attributable to CAP varies widely between countries and with the site of patient management. Robust national epidemiology monitoring for CAP is largely missing across Europe, with only Finland, Spain, and the UK having reliable epidemiological data. In the former socialistic countries of the Central and Eastern Europe (CEE) the epidemiology data on CAP are usually collected along with many other diseases and predominantly from hospital settings. Further, there is a lack of information on non-hospitalized CAP in the region. The objective of this study is to estimate and describe the incidence rates, mortality, and cost of CAP cases, in both inpatient and outpatient settings, in four CEE countries.

## Methods

### Ethics Statement

Data were not collected directly from patient charts by investigators and no identifying data about specific patients were collected. Therefore, informed consent was not necessary.

### The overall data integration approach

Data were collected on cases of hospitalized CAP treatment patterns, hospitalization rates, mortality, and treatment costs in CZ, PL, and SK based on national epidemiology reports mandatorily collected by governmental organizations. In Hungary, data were obtained from the National Health Insurance Fund [16], based on reimbursement claims. Generally, our aim was to utilize data collected within national surveillance programs among countries that impose a mandatory obligation for hospitals to report all hospitalized CAP cases. This approach was then supplemented by retrospective patient chart reviews conducted in the Czech Republic and Slovakia in 2010, and in Poland from 2007 to 2009[17] and from 2010 to 2011, with the aim to estimate the hospitalization rate and collect the resource use data for cost estimation. In Poland the costs of CAP were obtained from the valid DRG (Diagnosis Related Group) lists as reported by Jahnz-Rózyk (2010).[17]

In order to provide more detailed insight into the age-dependent disease burden, the elderly group was split into strata of 50–64, 65–74, 75–84 and ≥85 years of age. The size of the respective populations aged 50 years and older was obtained from the Statistical Office of each respective country for the latest available calendar year: 2010 (CZ); 2009 (PL, HU) and 2008 (SK). These age-specific population size figures were used to calculate the incidence of the CAP per 100,000 inhabitants by the specified age groups.

### The retrospective patient chart reviews

To enhance the incomplete mandatory CAP records in CZ and SK to include an estimate of outpatient CAP cases, and to estimate the direct and indirect costs, a retrospective survey was conducted with four types of physicians - the general practitioner, respiratory specialist, internal medicine specialist, and other specialist. These doctors were requested to provide retrospective patient chart data for a maximum of 10 all-cause CAP patients examined by the doctor in the period since January 1^st^ 2010 until the date of interview. Qualifying cases, regardless whether hospitalised or not, were those diagnosed with pneumonia, ICD10: J12–J18, based on examinations per current guidelines. To assure unbiased enrolment, patients were requested to be selected by date of the survey, starting with the most recent patient and proceeding to the past. The only inclusion criterion were age ≥50, CAP of any of the above given codes as a primary diagnosis, and complete medical chart record for the episode. Reported visits were allowed to be inpatient as well as outpatient; first or subsequent – after preceding referral. Prospective referrals were reported with specification to what specialist the patient was referred to. Each patient record encompassed the age, socio-economic status (employed, unemployed, retired), aetiology test used and result, the previous and subsequent outpatient visits, hospitalization, first and second line ATB medication, other medication, duration of hospitalization, outpatient and inpatient medical examinations and treatments, duration of sick leave where relevant (non-retired patients), and mortality status (survived pneumonia, died due to pneumonia). The obtained data were applied to investigate the hospitalization frequency.

A similar survey was conducted in Poland, evaluating data from 216 patients from the Department of Respiratory Medicine and Intensive Care (ICU) at the Military Institute of Medicine and from one primary health care facility in Warsaw in the period from October 1^st^ 2010 to September 30^th^ 2011.

No retrospective patient chart study was conducted in Hungary as the data for inpatient cases, outpatient cases, mortality, and treatment costs were obtained directly from the National Health Insurance Fund, within regular reports available publicly or upon request.

### Calculation of incidence and case fatality rate

In the Czech Republic, the incidence and case fatality rate by age groups of the inpatient CAP were calculated from the number of cases and fatalities reported by hospitals to the Institute of Health Information and Statistics of the Czech Republic. The incidence per 100,000 was then calculated versus the matching population size of the year 2010.

In Slovakia, the national figures on inpatient all-cause CAP incidence were obtained from the National Health Insurance Fund (NHIF) case report, covering years 2005 to 2009. The data included all cases coded under the following ICD-10: J12–J18, J85, J86. The data were stratified into age groups 45–54, 55–64, 65–74, 75–84, and 85+. In order to obtain age stratification the data were fitted with exponential regression model through the midpoint of each provided age stratum. The figures on inpatient all-cause CAP case fatality were obtained from the Statistical Office of the Slovak Republic case report, covering years 2005 to 2009.

In Poland, the numbers of inpatient CAP cases were retrieved for age groups 50–64, 65–74, 75–84, and 85+ from the Hospitalization prevalence study data report [13], [18]. The most recent report on the annual number of fatal cases for CAP could be obtained from the 2010 yearbook of the Central Statistical Office [19]. This data, however, reported the number of fatal cases of pneumonia in 2008 (herein denoted as D2008). As the number of new cases of CAP provided by The National Institute of Public Health – National Institute of Hygiene was from 2009 (denoted as C2009), a linear adjustment of the CFR for 2008 (denoted as CFR2008) proportional to the difference in total populations between 2008 and 2009 (denoted P2008 and P2009 respectively) had to be done, assuming the same incidence in 2008 as in 2009. The calculation was as follows:
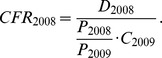



The outpatient CAP incidence was estimated using the outpatient-to-inpatient pattern obtained locally from three centres [17] and arranged to required age-groups upon request submitted to the author.

In Hungary, the national figures on the all-cause CAP incidence and fatality from the National Health Insurance Fund for the years 2006 to 2010 for inpatients and outpatients could be used to calculate the incidence per 100,000 directly by matching the data with the population size of the same year. The arithmetic average of the last three years was used to assure a more robust estimate. Similarly, the case fatality rate was estimated cumulatively for the three years period and expressed per one year by dividing by three.

### Calculation of direct and indirect costs

In the Czech Republic, estimates of the inpatient and outpatient care costs for CAP episode were based on the sum of costs for a hospital stay (only for inpatient costs) and outpatient visits as well as all reported medication, medical treatments, and medical examination costs. The most recent national reimbursement lists were used to assign costs to each item. Four types of doctors discussed previously were distinguished to set the outpatient costs.

No data from an analysis of the cost of CAP in adult patients have been available in Slovakia. However, due to common history and economic development, Slovakia and the Czech Republic have very similar health care systems. Therefore costs from an analysis conducted in the Czech Republic were applied both in Slovakia and Czech Republic.

In Poland, the direct cost could be obtained from a recently published source detailing the costs for elderly outpatient and inpatient CAP.[17] Within the study published, data were collected retrospectively from 2007 to 2009 in three different sites: a general practitioner family clinic for outpatient data and two hospitals in Warsaw for inpatient data. A micro-costing calculation method was used to estimate the outpatient costs. Inpatient costs were quantified using the Ministry of Health payment for each diagnosis group (DRG) added to each patient's inpatient treatment costs that are added to the cost of hospital stay.

In Hungary the direct costs of inpatient and outpatient pneumonia were obtained from the NHIF case reports requested by CEEOR. Direct costs of pneumonia were reported by NHIF for the years 2006 to 2010, in total from 155,887 inpatient cases and 652,918 outpatient cases, encompassing the same ICD codes as for incidence and fatality (J12, J13, J14, J15, J16, J17, J18). The average costs for each defined age group and each year could be calculated. As the final result, the average over the last three years (2008–2010; 2010 costs for outpatient CAP in order to match data collected in patient survey in CZ and SK) is given.

The indirect costs in all four countries were assumed to consist of the sick leave costs and hence they were assumed to only incur in the group 50–64 years of age. Daily salary was calculated from average salary reported by authorities in each state. Sick leave costs were then calculated using the human capital method [20] as:
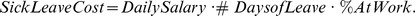



All costs were converted to Euros using rate EUR 1 = CZK 24.96; PLN 4.37 and HUF 304.67.

## Results

**Figure 1:**
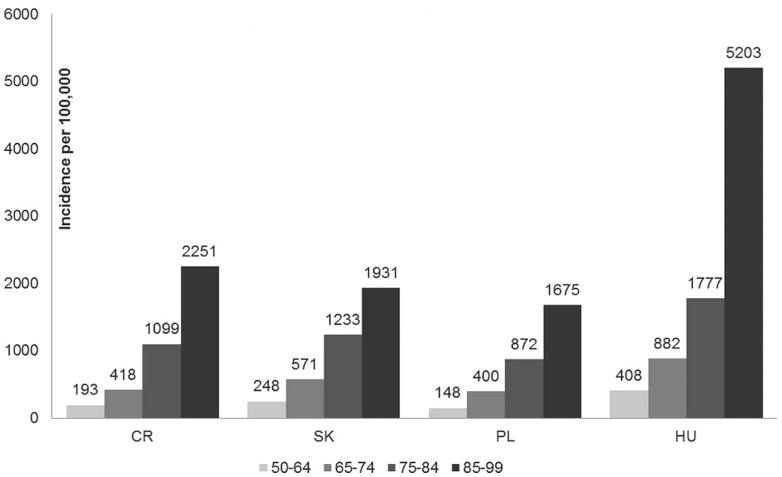
Country-specific incidence of all-cause inpatient CAP by age group per 100,000. National mandatory reports as available in CZ, SK and PL were used. In HU the incidence could be obtained from the reimbursement claims available at the National Health Insurance Fund.

The total number of hospitalizations (all cause CAP) among adults ≥ 50 years was: 18,003 (CZ); 9,260 (SK); 48,573 (PL); and 30,306 (HU), for an incidence rate per 100,000 person years of: 456.6 (CZ), 504.6 (SK), 363.9 (PL), and 845.3 (HU). Cumulatively for all four countries, compared with adults 50–64 years of age, the incidence of hospitalized CAP was 2.3 fold higher in those 65–74, 5.2 fold higher in 75–84, and 10.8 fold higher in those ≥85, manifesting an exponential trend. The incidence of hospitalized CAP for all adults ≥ 65 was a little less than twice that of adults ≥ 50 years (Table 1, Figure 1).

**Table 1:** Disease characteristics of hospitalized and outpatient CAP (all cause) by countries and age group.

	COMMUNITY ACQUIRED PNEUMONIA IN ELDERLY ADULTS							
	≥ 50 YEARS				≥ 65 YEARS			
	CZ	SK	PL	HU	CZ	SK	PL	HU
**Population size**	3,811,565	1,785,975	13,281,964	3,642,703	1,665,595	700,901	5,184,564	1,589,248
**CAP hospitalizations**	18,003	9,260	48,573	30,306	13,869	6,572	36,595	22,470
**CAP outpatient visits**	11,442	10,489	41,918	121,892	6,159	5,405	22,938	56,813
**Hospital deaths**	3,915	1,940	9,022	5,403	3,522	1,671	7,824	4,619
**Hospitalized incidence**	472	518	366	832	833	938	706	1,414
**Outpatient incidence**	300	587	316	3,346	370	771	442	3,575
**Hospital mortality (%)**	22	21	19	18	25	25	21	21

In the retrospective chart review study in the Czech Republic and Slovakia we received chart data from 573 patients (N_CR_ = 258; N_SK_ = 315) and estimated the hospitalization rate as 40%, 51%, 67%, and 84% for the age groups 50–64, 65–74, 75–84, and ≥85 respectively. The hospitalization rates were similar in the two countries. Estimating from the rate of hospitalization, the outpatient CAP incidence per 100,000 could be estimated for the respective age groups as 246, 343, 440, and 304 in CZ, and 469, 723, 863, and 736 in SK. In PL, the retrospective patient chart review supplied 198 patients, only 18 of which could be used to estimate the hospitalization rate. The overall hospitalization rate for all those ≥ 50 years of age was estimated as 8.72%. From these data, the incidence of outpatient CAP per 100,000 in PL was extrapolated as 1,697; 4,589; 10,008; and 19,213, respectively. In Hungary, where the primary case reports were based on reimbursement claims held by the NHIF, the incidence per 100,000 was estimated at 3,169; 3,484; 3,549; and 4,509, respectively, for the given age groups.

**Figure 2:**
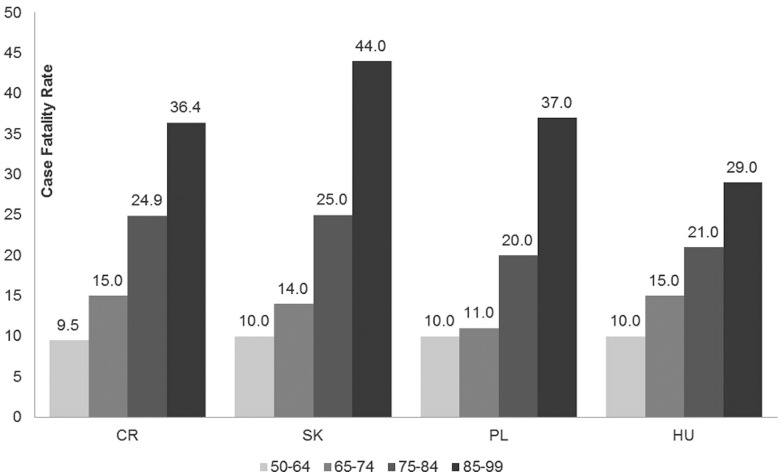
Country-specific case fatality rate per 100 hospitalized CAP cases by age group. The mortality was estimated using the retrospective patient chart in CZ, SK and PL and directly retrieved from the reimbursement claims available at the National health Insurance Fund in Hungary.

The fatality rate for hospitalized pneumonia in adults ≥ 50 years in the studied region is 19.1%, and given specifically for each country; 21.7% (CZ), 20.9% (SK), 18.6% (PL), 17.8% (HU). The case fatality rate was rather similar in all four countries ranging from largely consistent 10% in the age group 50–64 among all four countries up to 44% in those ≥85 and older in Slovakia (Figure 2).

The direct costs for CAP hospitalization ranged from 432 € (50–64, PL) to 1,045 € (50–64, SK) per case. Direct costs of outpatient cases were less than 10% of the cost of hospitalization, ranging from 4 € (50–64, HU) to 71 € (50–64, CZ). Within each country, direct costs were similar regardless of age. The direct costs of hospitalized patients ≥50 years were up to eight times higher than indirect costs (SK, PL, HU), while indirect costs were up to 40-times higher than direct costs in outpatients (HU; Table 2). The total economic burden of CAP in adults over 50 years of age was € 12,579,543 (CZ); 9,160,774 (SK); 22,409,085 (PL) and 18,298,449 (HU) per year.

**Table 2:** Costs per case of hospitalized and outpatient CAP (all cause) by countries and age group.

		COMMUNITY ACQUIRED PNEUMONIA IN ELDERLY ADULTS							
		50–64 YEARS				≥ 65 YEARS			
		CZ	SK	PL	HU	CZ	SK	PL	HU
**Hospitalized**	**Direct costs**	636.04 €	1,044.70 €	431.54 €	597.41 €	706.46 €	975.18 €	431.54 €	597.04 €
	**Indirect costs**	437.21 €	434.68 €	220.71 €	280.45 €	– €	– €	– €	– €
	**Total**	1,073.24 €	1,479.38 €	652.25 €	877.86 €	706.46 €	975.18 €	431.54 €	597.04 €
**Outpatient**	**Direct costs**	71.04 €	63.70 €	42.58 €	4.44 €	68.15 €	60.77 €	42.58 €	4.95 €
	**Indirect costs**	352.62 €	268.90 €	220.71 €	353.61 €	– €	– €	– €	– €
	**Total**	423.66 €	332.60 €	263.29 €	358.06 €	68.15 €	60.77 €	42.58 €	4.95 €

## Discussion

We estimated the incidence, case fatality rates (CFR), and economic burden of CAP in adults ≥50 years of age in the Czech Republic (CZ), Hungary (HU), Poland (PL) and Slovakia (SK) using a combination of administrative data and data from patient charts.

In general, our findings in four CEE countries support an incidence of pneumonia with similar age-dependent trend as reported from other countries.[13], [18], [21] Gil-Prieto et al investigated the incidence of hospitalized pneumonia using administrative data in Spain and reported rates of 627/100,000 persons >50 years of age and 1,029/100,000 in persons >65, which compares to ranges of 366 – 832 and 706 – 1414, respectively, across the four countries in our study. Froes et al reported rates in Portugal of 196, 592, 1694, and 4430 per 100,000 in persons 55–64, 65–74, 75–84, and 85+ years of age, respectively. In Germany, hospitalized incidence was estimated at around 286 per 100,000 inhabitants, with a steeply increasing incidence with advancing age (data from 2005–2006) [21]. In the USA, an incidence of 18.2 cases per 1,000 person-years among persons aged 65–69 years was reported in 2006. In older adults aged ≥85 it was as high as 52.3 cases per 1,000 person-years [12] [13]. In a Finnish study, the incidence of CAP rose dramatically with age, with a six-fold increase in incidence between ages 30–44 years and ≥75 years [18]. In-hospital mortality was around 14% and exceeded 20% in those older than 80 years [21]. In all cases, the rates were generally higher than that from CZ, SK, and PL but lower than HU. The rate of increased incidence with increasing age was similar in the studies.

There are few studies comparing inpatient and outpatient incidence rates. In the US, Jackson et al. (2004) reported incidence of 11.5 hospitalized cases and 16.8 outpatient cases per 1000 person-years among persons ≥65 years of age for a hospitalization rates of 40.7%. The likelihood of hospitalization increased from 26.7% in persons 65–69 to 60.1% in persons ≥90. With the exception of HU, a greater proportion of CAP cases were hospitalized in four CEE countries in our analysis. However, the ratio of inpatient to outpatient admissions is quite consistent with that observed in other studies where increasing age is associated with increasing likelihood of inpatient admission.

The mortality we observed in hospitalized patients in the four CEE countries is roughly twice as high as in the western part of Europe [18],[22]–[25]. To be precise, the rates for all cause hospitalized CAP are 25% in CZ, 25% in SK, 21% in PL and 21% in HU, respectively, in those aged ≥65 (Figure 2). For comparison, 10.3% to 14% mortality was reported in Germany [21] [22], 11.6% in Spain [23], 13.8% in Italy [24] and 11.5% in Ireland [25], for the same age group ≥ 65 years in cases identified by ICD-9 codes from European hospital databases, and therefore are not too dissimilar from our approach. A Finnish study reports mortality of 11% in those ≥ 60 years [18]. In a recent comprehensive European review the mortality varied between <1% to 48% and was associated with advanced age, co-morbid conditions and CAP severity [13]. CAP mortality was shown to differ differs in upper income and middle income regions. In a recent cohort study with 6371 conferred CAP patients 18 years of age and older, mortality was found significantly different between US/Canada (7.3%), Europe (9.1%), and Latin America (13.3% in total, excess of 35% in PSI risk class V) [26]. At least 13% of patients in the Latin America region were <50 years of age, based on the proportion in PSI risk class I, so overall CAP mortality in persons over 50 was at least 14%, excluding the risk class I proportion, and probably higher if all <50 patients could be excluded. Our finding in the 50–64 age group is not dissimilar from that reported elsewhere (Figure 2). Generally, the high average mortality in our study is driven by those older than 75 years of age. A possible reason for the increased mortality may be somewhat specific approach in use of antibiotics, mainly aminopenicillins, along with increased concern of developing resistance that prevents more aggressive therapeutic approach such as use of respiratory quinolones. Generally, there are many potential sources of differences between countries. For example, the threshold of admission to the hospital may differ such that sicker patients are more or less likely to be admitted. Lower standards of care, poorer general comorbid health of the population, and/or other environmental risk factors may also drive higher fatality. Finally differences in coding practices may result in a greater proportion of nosocomial or health care associated pneumonia being included in the sample. However, the coding applied in our study as CAP was similar to that applied in the above comparisons.

In all countries where no mandatory system for reporting all cases of CAP exists, it is difficult to estimate the incidence of outpatient CAP without conducting a thorough prospective study with rigorous definition of the disease cases to be enrolled. In this paper we made an effort to estimate, within the overall burden of this disease, the contribution of ambulatory cases. Since outpatient pneumonia cases are often not collected by national databases, and therefore frequency of such cases is often unknown, the major limitation of this effort was the heterogeneous character of the sources used in each country. The outpatient CAP percentage in the total CAP number was therefore quantified using the retrospective survey of patient charts. Then the overall incidence in those ≥50 years of age in CZ, SK and PL as well as the incidence by age groups in each country could be estimated, based on the known inpatient CAP number. As for Hungary, the National Health Insurance Fund is the only source of data in the country. While the majority of CAP among adults 50–64 years of age was treated outpatient, the proportion of CAP hospitalized increased with increasing age. The pattern of outpatient CAP visits differ country to country for variety of reasons, which may include disease coding, imperfect reporting, disease definition, and technical limitation of diagnostics, etc. The differences in reporting and source of data may be the reason for much higher incidence rates and mortality in Hungary, where incidence rates were ten times higher than in the rest of the countries in the analysis.

Hospitalization represents over 90% of the direct costs of treatment in all four countries. Adults aged ≥65, who represent 41% of the combined population, account for 73% of the costs. The costs per case remain relatively stable both for inpatient and outpatient CAP across all age groups, but we observed the total costs in the population increasing with age for hospitalized population as a function of the increase in incidence and population size, so as to reach a maximum cost at age group 75–84. By contrast, the overall cost of outpatient care declined with age since the incidence was generally steady and population sizes were larger in the younger groups.

In this study we were limited to identification of pneumonia cases through ICD-10 registries, which are general codes that may include unconfirmed cases, nosocomial pneumonias, readmissions, or hospitalizations due to other causes [27]–[31]. To the extent that nosocomial pneumonias or healthcare associated pneumonias are coded as principle diagnosis, this may overstate incidence rates or mortality of CAP. When ICD-coded all-cause hospitalized pneumonia data have been compared against clinical records to identify radiographically confirmed community acquired pneumonia in US adults, between 54%–71% of coded cases meet these criteria [27]–[31]. Coding practices may vary by country and region, and similar data are not currently available from CEE countries, therefore the transferability of these data is uncertain. Nevertheless, our approach provides a useful and consistent benchmark for evaluating disease trends across a population and over time that may facilitate health policy planning and evaluation [32]. Further research into validation of administrative coded pneumonia cases in CEE countries with regard to cause and aetiology would significantly benefit our understanding of the nature of pneumonia in adults.

Further research into the etiology of pneumonia and risk profile of the population could help guide prevention efforts in the area. The presence of comorbidities such as cardiovascular disease, chronic pulmonary disease, and diabetes can increase pneumonia risk among elderly persons, and management of related risk factors may reduce the risk of pneumonia hospitalizations and mortality. Increasing use of influenza and pneumococcal vaccination in the elderly and persons at risk could also play a role in reducing the burden of pneumonia hospitalizations.

Elderly adults compose a significant portion of the population in the Central European countries with a steadily growing trend as birth rates decline and life expectancy increases. Inhabitants 50 years of age and older represent 35% of the total population in the studied region with only slight differences; 37% (CZ) 33% (SK), 35% (PL) 37% (HU). Among adults ≥50 years of age, the elderly ≥65 compose 41% in the region, with country-specific percentages of: 44% (CZ); 39% (SK); 39% (PL) and 44% (HU). Comorbidities are more common among the elderly, contributing to both increased risk of disease and poorer outcome. With the increases projected in the elderly population [18], the absolute burden of disease is likely to increase, putting greater pressure on healthcare systems. It's imperative that therapeutic and preventive interventions be developed to address the specific pathogens causing CAPs, as well as the spread of antibiotic resistance, to curb the growth of this disease burden.
